# Hyperglycemia and Blood Pressure Treatment Goal: A Cross Sectional Survey of 18350 Patients with Type 2 Diabetes in 77 Tertiary Hospitals in China

**DOI:** 10.1371/journal.pone.0103507

**Published:** 2014-08-14

**Authors:** Linong Ji, Xinyue Zhi, Juming Lu, Xiaohui Guo, Wenying Yang, Weiping Jia, Dajin Zou, Zhiguang Zhou, Qiuhe Ji, Dalong Zhu, Lixin Shi, Jianping Weng

**Affiliations:** 1 Department of Endocrinology, People’s Hospital of Peking University, Beijing, China; 2 Department of Epidemiology and Biostatistics, School of Public Health, Tianjin Medical University, Tianjin, China; 3 Department of Endocrinology, The General Hospital of Beijing Military Command, Beijing, China; 4 Department of Endocrinology, First Hospital of Peking University, Beijing, China; 5 Department of Endocrinology, Sino-Japan Friendship Hospital, Beijing, China; 6 Department of Endocrinology, Shanghai Sixth People’s Hospital, Shanghai Jiaotong University, Shanghai, China; 7 Department of Endocrinology, Changhai Hospital of Shanghai, Shanghai, China; 8 Department of Endocrinology, The Second Xiangya Hospital of Central South University, Changsha, China; 9 Department of Endocrinology, Xijing Hospital Affiliated to 4th Military Medical University, Xi’an, China; 10 Department of Endocrinology, Nanjing Drum Tower Hospital, Nanjing, Jiansu, China; 11 Department of Endocrinology, Hospital of Guiyang Medical University, Guiyang, China; 12 Third Affiliated Hospital of Sun Yat-sen University, Guangzhou, China; Central China Normal University, China

## Abstract

**Objective:**

To investigate the association of hyperglycemia with blood pressure control goal in the patients with type 2 diabetes (T2D) cared by tertiary hospitals in China.

**Study Design and Methods:**

A cross sectional survey of 29442 patients was conducted in 77 tertiary hospitals in 4 major cities in China in 2011 and 18350 of them without known hypertension were used in the analysis. Univariable and multivariable logistic regression analysis stratified on cities and hospitals was performed to obtain odds ratio of factors of interest for achievement of the blood pressure treatment goal, i.e., 140/80 mmHg as recommended by American Diabetes Association (ADA). Sensitivity analysis was performed after re-inclusion of 11902 patients with diagnosed hypertension. Findings from were further replicated in patients with T2D recruited using the same protocol from tertiary hospitals located in other central cities in China.

**Results:**

The mean age was 58.2 (SD: 11.3) years and 53.3% were male, with a median of 4 years of disease duration. A total of 12129 patients (58.2%) did not achieve the ADA recommended goal for BP control. After adjusting for covariables, hyperglycemia was associated with failure to achieve the BP goal (OR of HbA1c at 6.5%–6.9% vs. <6.0%: 1.22, 95%CI: 1.08 to 1.39; OR of 7.0%–7.0% vs. <6.0%: 1.37, 1.21 to 1.54 and OR of ≥8.0% vs. <6.0%: 1.22, 95%CI: 1.08 to 1.38). The sensitivity analysis and the replication analysis showed similar results.

**Conclusions:**

Hyperglycemia defined as HbA1c≥6.5% increased the risk of failure to achieve the BP goal in T2D patients.

## Introduction

Diabetes has been increasing in the world, especially in Asian countries including China [1], [2]. It predisposes to increased risk of microvascular and macrovascular diseases [3] and cancer [4], [5]. Hypertension occurs in up to 30–40% of patients with type 2 diabetes (T2D) [6], [7] and itself is a risk factor for cardiovascular disease and renal disease in both general population and diabetic population [8], [9]. In the United Kingdom Prospective Diabetes Study (UKPDS) coronary heart disease risk engine, systolic blood pressure (SBP) was an independent predictor for coronary heart disease among patients with T2D [10]. The Steno-2 Study has demonstrated that a multifactorial intervention protocol with use of multiple drugs to control hyperglycemia, hypertension and high low-density lipoprotein cholesterol (LDL-C) can safeguard patients from developing vascular complications and from death due to any cause and cardiovascular causes [11]. Although further tight control of systolic blood pressure (SBP) below 120 mm Hg does not result in a further reduction in cardiovascular disease as compared with the standard SBP target of 140 mmHg [12], achievement of good control of hyperglycemia, high blood pressure and abnormal lipids plays a crucial role in clinical management of T2D [13].

T2D is characterized by relative or absolute shortage of insulin secretion and hyperglycemia, and hyperglycemia control plays a fundamental role in management of T2D. In this regard, the United Kingdom Prospective Diabetes Study [14], [15] showed that maintaining glycated hemoglobin (HbA1c) around 7% by intensive blood-glucose control as compared to 7.9% in the conventional group was able to achieved a 25% risk reduction in microvascular endpoints over a 10-year period and a 24% risk reduction in the microvascular endpoint and 15% risk reduction in myocardial infarction over further 10 years of follow-up [6]. On the other hand, the Action to Control Cardiovascular Risk in Diabetes (ACCORD) trial found that tight control of HbA1c below 6.0% increased mortality risk [16], and both the ACCORD trial and the Veterans Affairs Diabetes Trial (VADT) did not find that tight control of HbA1c below 6.0% led to an additional reduction in the risk of cardiovascular disease [16], [17]. However, the Action in Diabetes and Vascular Disease Preterax and Diamicron Modified Release Controlled Evaluation [9], [11] did suggest that achievement of HbA1c below 6.5% was able to further reduce nephropathy by about 20%, which may be translated to a CVD risk reduction in the long run [18]. Obesity, hypertension and insulin resistance often occur in clusters to increase the risk of diabetes [19], [20], [21]. On the other hand, hyperglycemia has potent but reversible oxidizing effects on LDL-particles [22], resulting in increased oxidative stress which may activate renin-angiotensin system via cross-talks [23], [24], leading to increased blood pressure. Thus, a biological link between hyperglycemia and BP in T2D is plausible. Nevertheless, it remains uncertain how tight hyperglycemia control is good enough not to increase blood pressure, or to achieve the treatment target among patients with T2D as recommended by the American Diabetes Association (ADA), i.e., systolic/diastolic BP<140/80 [13].

This study used a large cross sectional survey of 29442 patients with T2D in 77 tertiary hospitals in China to address the association between hyperglycemia control and non-achievement of the ADA’s BP treatment goal and in particular, whether hyperglycemia at 6.5%–6.9% was associated with increased risk of failure to achieve the ADA’s BP treatment goal among Chinese patients with T2D under tertiary care.

## Research Design and Methods

### Patients

The Chinese Diabetes Association launched an HbA1c surveillance system among patients with T2D in the mainland China in 2009. A total of 400 hospitals from 75 cities in 20 provinces, 3 autonomous regions and 4 municipalities (Beijing, Shanghai, Tianjin and Chongqing) directly under the central government agreed and participated in the surveillance system. The number of participating hospitals was increased to 414, with 81 cities in 30 provincial administrative regions of China in 2011, from all the provincial administrative regions in China except for Tibet. The ethics approval was obtained from the Ethics Committee of Chinese PLA General Hospital and written informed consent was obtained before collecting data from the patients. The survey in 2011 was conducted from March to June 2011. The inclusion criteria were: 1) being an outpatient with T2D treated with OADs alone, OADs combined with insulin, or OADs combined with GLP-1 receptor agonists; Aged 18 years and more; 2) with at least one previous outpatient medical record pertaining to diabetes; being a local resident for at least 6 consecutive months prior to participation in the study. The exclusion criteria included: 1) diabetes secondary to other diseases; 2) on insulin monotherapy; 3) not on OAD monotherapy, OADs combined with insulin, or OADs in combination with GLP-1 receptor agonists; 4) Type 1 diabetes; 5) inpatients; 6) on diet and other lifestyle therapy only or on Chinese herbal medicine; 7) being pregnant or breast-feeding an infant; 8) being unable to complete the survey due to mental diseases; and 9) unconsciousness or being unable to communicate.

During the recruitment period, health professionals (junior doctors, nurses or postgraduate medical students) sequentially screened patients with type 2 diabetes for their eligibility. Those who met the inclusion criteria and did not have any of the exclusion criteria were invited to participate in the survey. The process continued until 7 patients were successfully recruited in a consecutive way in each day and until 400 patients were recruited in the pre-specified period. After obtaining informed consent, the health professional/s reviewed the medical notes including the results of laboratory essays and recorded the related data in a form. The retrieved data included gender, height, and weight, and blood pressure and date of diagnosis of diabetes. Laboratory data on HbA1c, and lipid profile were recorded. Specific information about the treatments used for the management of their T2D was documented, including the use of OADs (including DPP-4 inhibitors and GLP-1 receptor agonists), and different types of insulin, as well as combinations of these antidiabetes drugs. A special staff member entered all the data and uploaded the entered data to the central database.

This analysis chose to analyze data of subjects recruited from the accredited 3A hospitals in four well-developed cities in China: Beijing, Shanghai, Tianjin and Guangzhou. The 3A hospitals were the best hospitals that are assumed to provide quality care to T2D patients. The reason to select 3A hospitals in four cities was that the coverage rates of the 3A hospitals were high, and may be assumed preventative of the population of patients with T2D cared by the top hospitals in the cities concerned. The coverage rates of the 3A hospitals were 74.4% for Beijing (n = 32), 76% for Shanghai (n = 22), 55% for Tianjin (n = 11) and 29.3% for Guangzhou (n = 12) after excluding those 3A hospitals that recruited less than 30 patients during the pre-specified recruitment period. The recruitment goal was 400 patients by each hospital.

A total of 29442 patients were recruited in 77 Tertiary Hospitals in China were successfully recruited and used in the analysis for this study, in which 18350 patients with T2D but without known hypertension were used in the analysis.

### Clinical outcomes

Prior coronary heart disease, cerebrovascular disease, diabetic retinopathy, diabetic neuropathy, diabetic nephropathy, diabetes-related foot ulcers, and others diagnosed by secondary care hospitals or tertiary hospitals were retrieved from medical notes, including dates of diagnosis of these medical conditions. Prior hypertension was defined as previously having systolic/diastolic BP≥140/90 mmgHg or on antihypertensive drug treatment before the index visit.

### Statistical Analysis

The Statistical Analysis System (SAS) release 9.3 (SAS Institute Inc., Cary, NC, USA) was used in all the data analysis. Data were expressed as mean (standard deviation, SD) if normal distributions could not be rejected or median (25^th^ to 75^th^ percentile) if normal distribution was rejected by checking the Q-Q plot of the variable concerned. Chi-square test or Fisher’s exact test where appropriate was used to compare categorical variables and Student t test or Wilcoxon Two Sample test where appropriate was used to compare continuous variables between two groups. We used the ADA’s recent recommendation to define failure to achieve the BP treatment goal, i.e., ≥140/80 mmHg [7], and defined hyperglycemia into 5 groups according to HbA1c levels, i.e., HbA1c<6.0%, 6.0% to 6.4%, 6.5 to 6.9%, 7.0% to 7.9% and ≥8.0%. Body mass index (BMI) was calculated as body weight in kilograms divided by squared body height in meters. Duration of diabetes was calculated as the period from the date of diagnosis of diabetes to that of measurement of HbA1c. Logistic regression analysis was used to obtain odds ratio [15] of factors of interest for non-achievement of the BP treatment goal in univariable and multivariable analysis. Use of stratified logistic models on cities and hospitals was to adjust for the effect of differences in the number of patients recruited by different cities and hospitals. A p value below 0.05 for two-sided tests was considered as statistically significant.

Sensitivity analysis was performed after re-inclusion of 11902 patients with diagnosed hypertension. The key findings from the analysis above were further replicated in patients with T2D recruited using the same protocol from 3A hospitals located in other regional central cities, i.e., Chengdu (Southwest China, the coverage rate: 50.0%, n = 9) of the 3A hospital in that Xi’an (West China, the coverage rate: 13.0%, n = 3), Wuhan (East China, the coverage rater: 40.0%, n = 10) and Shenyang (Northeast China, the coverage rate: 70.6%, n = 12) to show the consistency in Chinese patients with T2D under management of 3A hospitals in other regions, China.

## Results

### Characteristics of the patients

The mean age of the T2D patients in the analysis was 58.2 (standard deviation [SD]: 11.3) year, with a median duration of diabetes mellitus of 4.0 (25^th^ to 75^th^ percentiles: 2.0–9.0) years. A total of 12129 patients (58.2%) did not achieve the ADA recommended goal for BP control, with a significant difference by city, ranging from 50.6% in Guangzhou, 61.6% in Beijing, 73.6% in Shanghai to 83.8% in Tianjin. There were more male patients than female patients (53.5% vs. 46.5%, p<0.0001). The patients who did not achieve the BP goal were more likely to be male, had higher body height, higher BMI, and had longer duration of diabetes and worse glucose and lipid metabolism than those patients who achieved the goal (Table 1).

**Table 1:** Clinical and biochemical characteristics of 18350 patients with Type 2 diabetes and with diagnosed hypertension.

	BP goal not achieved	BP goal achieved	
	(n = 12129)	(n = 6221)	
Variables	Mean/number (SD or %)	Mean/number (SD or %)	*P* value
Age, year	58.2(11.2)	58.1(11.4)	0.2071
Male gender	6825(56.5%)	2962(47.6%)	<0.0001
Body height, cm	166.8(7.9)	165.1(7.6)	<0.0001
BMI, kg/m^2^	24.3(3.1)	23.9(3.2)	0.0063
BMI groups, kg/m^2^			<0.0001
Overweight	5562(45.9%)	2280(36.7%)	
Obesity	1066(8.8%)	484(7.8%)	
Duration of diabetes, year	6.8(6.2)	5.4(4.8)	<0.0001
Duration of diabetes groups, year			<0.0001
<1 year	1830(15.1%)	1107(17.8%)	
<1–2.9 years	2893(23.9%)	1883(30.3%)	
3–5.9 years	2554(21.1%)	1448(23.3%)	
6–9.9 years	2342(19.3%)	896(14.4%)	
10 years and above	2510(20.7%)	887(14.3%)	
HbA1c, %	7.7(1.5)	7.6(1.7)	<0.0001
HbA1c groups, %			<0.0001
<6.0	776(6.4%)	604(9.7%)	
6.0 to 6.4	998(8.2%)	903(14.5%)	
6.5 to 6.9	1891(15.6%)	1038(16.7%)	
7.0 to 7.9	4120(34.0%)	1500(24.1%)	
≥8.0	4344(35.8%)	2196(35.0%)	
Low density lipoprotein cholesterol, mmol/L	3.00(1.09)	2.83(0.85)	<0.0001
Triglyceride, mmol/L	1.98(1.47)	1.68(1.19)	<0.0001
Total cholesterol, mmol/L	4.46(1.59)	4.65(1.28)	<0.0001
**Location**			
Beijing	2441(50.0%)	4054(44.6%)	
Tianjin	544(11.2%)	1221(13.4%)	
Shanghai	755(15.5%)	2436(26.8%)	
Guangzhou	1140(23.4%)	1379(15.2%)	

*, Median (25^th^ percentile to 75^th^ percentile) and their P values were derived from Two Sample Wilcoxon test.

### Hyperglycemia and the blood pressure control target

As compared to those who had a HbA1c level below 6.0%, patients who had HbA1c levels of 6.5%–6.9%, 7.0%–7.9% and ≥8.0% were all associated with higher risk of failure to achieve the BP control goal in univariable analysis. After adjusting for covariates including age, gender, BMI, duration of diabetes and home glucose monitoring, etc., hyperglycemia defined as HbA1c levels at 6.5%–6.9%, 7.0%–7.9% and ≥8.0% remained associated with increased risk of failure to achieve the BP goal (OR of HbA1c at 6.5%–6.9% vs. <6.0%: 1.22, 95%CI: 1.08 to 1.39; OR of 7.0%–7.0% vs. <6.0%: 1.37, 1.21 to 1.54 and OR of ≥8.0% vs. <6.0%: 1.22, 95%CI: 1.08 to 1.38) (Table 2).

**Table 2:** Hyperglycemia for blood pressure goal achievement in 18350 Chinese patients with Type 2 diabetes and without diagnosed hypertension.

Variables	Number (%)	OR (95% CI)	P Value
**Univariable model**			
HbA1c Groups, %			<0.0001
<6.0	998(52.5%)	Reference	
6.0 to 6.4	1891(64.5%)	0.94(0.80 to 1.11)	
6.5 to 6.9	4120(73.3%)	1.36(1.16 to 1.59)	
7.0 to 7.9	4344(66.6%)	1.59(1.37 to 1.86)	
≥8.0	776(52.2%)	1.38(1.19 to 1.61)	
**Multivariable model one**			
Age, per 10 years		1.06(0.83 to 1.10)	0.0005
Male vs. female		0.94(0.85 to 1.05)	0.2684
Body mass index, per kg/m^2^		1.05(1.04 to 1.06)	<0.0001
Duration of diabetes, per 5 years		1.05(1.01 to 1.10)	0.0200
Home glucose monitoring	4518(59.2%) vs. 7611(68.7%)	0.99(0.99 to 1.08)	0.8484
HbA1c Groups, %			<0.0001
<6.0	998(52.5%)	Reference	
6.0 to 6.4	1891(64.5%)	0.98(0.83 to 1.15)	
6.5 to 6.9	4120(73.3%)	1.33(1.13 to 1.56)	
7.0 to 7.9	4344(66.6%)	1.46(1.25 to 1.71)	
≥8.0	776(52.2%)	1.23(1.05 to 1.44)	
**Multivariable model two**			
Age, per 10 years		1.17(1.08 to 1.27)	0.0002
Male vs. female		1.06(0.96 to 1.18)	0.2636
Body mass index, per kg/m^2^		1.05(1.04 to 1.06)	<0.0001
Duration of diabetes, per 5 years		1.05(1.01 to 1.10)	0.0183
Self home glucose monitoring	4518(59.2%) vs. 7611(68.7%)	0.99(0.91 to 1.08)	0.7988
HbA1c≥6.5% vs. <6.5%	8464(69.7%) vs. 3665(59.0%)	1.17(1.08 to 1.27)	0.0003

Variables adjusted for in the multivariable analysis included age, gender, body mass index, body height, self home glucose monitoring and treatment schemes (one oral antidiabetes drug [OAD] only, two OADs only, three OADs only, four and more OADs, and OADs plus insulin).

### Sensitivity analysis and replication in other populations of patients with Type 2 diabetes

After re-inclusion of 11092 patients with known hypertension, the statistical significance for OR of HbA1c levels 6.5%–6.9% vs. <6.0%, 7.0%–7.9% vs. <6.0% and ≥8.0% vs. <6.0% remained (Table 3). In a similar way, the findings were replicated in patients with T2D recruited using the same procedure in other major regional cities: Chengdu, Xi’an, Wuhan and Shenyang (Table 4).

**Table 3:** Sensitivity analysis of hyperglycemia for blood pressure goal achievement in 18350 Chinese patients with Type 2 diabetes and without diagnosed hypertension and 11092 patients with Type 2 diabetes and diagnosed hypertension.

Variables	Number (%)	OR (95% CI)	P Value
**Univariable model**			
HbA1c Groups, %			<0.0001
<6.0	1770(59.9%)	Reference	
6.0 to 6.4	3183(69.4%)	1.02(0.89 to 1.15)	
6.5 to 6.9	6884(77.1%)	1.30(1.15 to 1.48)	
7.0 to 7.9	7899(73.4%)	1.55(1.37 to 1.74)	
≥8.0	1394(63.2%)	1.43(1.27 to 1.61)	
**Multivariable model one**			
Age, per 10 years		1.13(1.10 to 1.16)	<0.0001
Male vs. female		1.00(0.92 to 1.09)	0.9524
Body mass index, per kg/m^2^		1.07(1.06 to 1.08)	<0.0001
Duration of diabetes, per 5 years		1.06(1.02 to 1.09)	0.0005
Home glucose monitoring	8666(70.1%) vs. 12464(73.0%)	0.98(0.92 to 1.05)	0.5630
HbA1c Groups, %			<0.0001
<6.0	1770(59.9%)	Reference	
6.0 to 6.4	3183(69.4%)	1.01(0.89 to 1.15)	
6.5 to 6.9	6884(77.1%)	1.22(1.08 to 1.39)	
7.0 to 7.9	7899(73.4%)	1.37(1.21 to 1.54)	
≥8.0	1394(63.2%)	1.22(1.08 to 1.38)	
**Multivariable model two**			
Age, per 10 years		1.17(1.08 to 1.27)	0.0002
Male vs. female		1.06(0.96 to 1.18)	0.2636
Body mass index, per kg/m^2^		1.05(1.04 to 1.06)	<0.0001
Duration of diabetes, per 5 years		1.05(1.01 to 1.10)	0.0183
Self home glucose monitoring	8666(70.1%) vs. 12464(73.0%)	0.99(0.91 to 1.08)	0.7988
HbA1c≥6.5% vs. <6.5%	14783(75.1%) vs. 6347(65.1%)	1.17(1.08 to 1.27)	0.0003

Variables adjusted for in the multivariable analysis included age, gender, body mass index, body height, self home glucose monitoring and treatment schemes (one oral antidiabetes drug [OAD] only, two OADs only, three OADs only, four and more OADs, and OADs plus insulin).

**Table 4:** Replication of hyperglycemia for blood pressure goal achievement in 13689 Chinese patients with Type 2 diabetes and without diagnosed hypertension in other regional central cities in China.

Variables	Number (%)	OR (95% CI)	P Value
**Univariable model**			
HbA1c Groups, %			0.0008
<6.0	543(6.3%)		
6.0 to 6.4	731(8.5%)	0.93(0.71 to 1.23)	
6.5 to 6.9	1179(13.8%)	1.31(1.01 to 1.68)	
7.0 to 7.9	2605(30.4%)	1.26(0.99 to 1.60)	
≥8.0	3501(40.9%)	1.14(0.90 to 1.45)	
**Multivariable model one**			
Age, per 10 years		1.09(1.04 to 1.18)	0.0002
Male vs. female		1.16(101 to 1.34)	0.0353
Body mass index, per kg/m^2^		1.09(1.07 to 1.11)	<0.0001
Duration of diabetes, per 5 years		0.93(0.88 to 0.99)	0.0175
Home glucose monitoring	?	1.11(0.99 to 1.25)	0.0803
HbA1c Groups, %			0.0185
<6.0	543(6.3%)	Reference	
6.0 to 6.4	731(8.5%)	1.05(0.83 to 1.34)	
6.5 to 6.9	1179(13.8%)	1.33(1.07 to 1.66)	
7.0 to 7.9	2605(30.4%)	1.31(1.06 to 1.62)	
≥8.0	3501(40.9%)	1.27(1.03 to 1.57)	
**Multivariable model two**			
Age, per 10 years		1.09(1.04 to 1.15)	0.0002
Male vs. female		0.86(0.75 to 0.99)	0.0312
Body mass index, per kg/m^2^		1.09(1.07 to 1.11)	<0.0001
Duration of diabetes, per 5 years		0.94(0.89 to 0.99)	0.0215
Self home glucose monitoring		1.12(1.00 to 1.26)	0.0614
HbA1c≥6.5% vs. <6.5%	?	1.10(0.98 to 1.23)	0.0979

Variables adjusted for in the multivariable analysis included age, gender, body mass index, body height, self home glucose monitoring and treatment schemes (one oral antidiabetes drug [OAD] only, two OADs only, three OADs only, four and more OADs, and OADs plus insulin).

## Conclusion

Using a large survey of patients with T2D under the care of top tertiary hospitals in China, we are the first reporting that hyperglycemia defined as HbA1c at 6.5% to 6.9% increased the risk of failure to control high blood pressure below the treatment goal [13]. The result is robust as suggested by the sensitivity analysis and the replication in Chinese patients with T2D under management of top tertiary hospitals in other regional major cities in China.

The UKPDS [6], [15] and DCCT [25], [26] demonstrated that hyerglycemia control to a level around 7% was able to reduce the incidence rates of micro- and macro-vacular diseases in T2D and Type 1 diabetes, respectively. However, the recent ADVANCE trial showed that further tight control of hyperglycemia below 6.5% was achievable and able to further reduce the incidence of micro-vascular disease in T2D [18]. On the other hand, it is less known whether tight hyperglycemia control below 6.5% is associated with decreased risk of hypertension in T2D and remained unknown that how tight control of hyperglycemia contributes to the high BP control although it is known that multifactorial intervention controlling hyperglycemia, hypertension and high LDL-C can reduce the risk of vascular diseases, all-cause death and cardiovascular death [11].

Although the biological links between exposure to hyperglycemia and micro- and macro-vascular diseases are complicated [17], [27], there are consistent reports confirming that hypertension confers an additional risk for macro-vascular diseases in both patients with and without T2D [2], [8], [18]. On the other hand, there are few studies addressing how tight hyperglycemia is tight enough to achieve the BP control goal. Our data show that 1) suboptimal glycemia control contributed to increased risk of failure to achieve the BP control goal; and 2) HbA1c level of 6.5% to 6.9% was still associated with increased risk of non-achievement of the BP treatment goal. The results are consistent with the epidemiological findings from a pooled analysis of nine studies from five countries with 44,623 participants that recommended a cutoff point of 6.5% for HbA1c for increased risk of diabetes-specific retinopathy and an alternative diagnostic criterion for diabetes [8].

There are several mechanisms have been proposed to explain the high frequency of hypertension in among patients with T2D [28], [29], including the stimulating effect of hyperinsulinemia on sympathetic drive, smooth muscle growth, and sodium–fluid retention and the excitatory effect of hyperglycemia on the renin–angiotensin system (RAS) [29]. In this regard, hyperglycemia increases tissue angiotensin II, which induces oxidative stress and endothelial damage [30]. A study reported that hyperglycemia has a potent but reversible effect on LDL oxidation [22]. Oxidized LDL then enhances the expression and activation of RAS components (18). Activated RAS, especially local RAS, may play a role in hypertension as well as dyslipidemia, glucose intolerance and insulin resistance [31]. Hence, a biological link between hyperglycemia and high BP is highly plausible.

The current clinical guideline of the American Diabetes Association for T2D is to reduce glycated hemoglobin (HbA1c) below the target of 7.0% [7]. The UKPDS demonstrated that a 10-year intensive glucose control by sulphonylureas or insulin achieved an 11% reduction by tight hyperglycemia control of HbA1c below 7.0% vs. the conventional care of HbA1c below 7.9%. Although the intensive hyperglycemia control did not lead to a significant risk reduction in the rate of macro-vascular disease over a 10-year intensive management, it did achieve a 9% reduction in the rate of any diabetes-related end point and risk reduction in microvascular disease. Further tight hyperglycemia control to a level of HbA1c<6.5% may lead to further reduction in the rate of micro- and macro- vascular complications in patients of type 2 diabetes. In this regard, the Outcome Reduction With Initial Glargine Intervention (ORIGIN) study [32] suggested further tight control of hyperglycemia can achieve an additional risk reduction in diabetes complications when HbA1c was lower than 6.5%, which is safe and able to reduce nephrology risk. The ADVANCE trial [9] demonstrated that reducing HbA1c further to <6.5% is safe and able to further reduce nephrology risk by about 20%. The reduction in albuminuria in the ADVANCE trial may be translated to future reduction in cardiovascular disease [18]. Thus, our data support the notion that intensive hyperglycemia control with a threshold of HbA1c below 6.5% contributes to good BP control in addition to reduced risk of microvascular disease [9], and potentially reduced risk of macrovascular disease in the long run [18].

This study has several limitations. Firstly, the study was a cross-sectional survey and can not establish a causal relationship between hyperglycemia and hypertension. Obesity, insulin resistance and hypertension often appear in clusters and metabolic syndrome is an established risk factor for diabetes [2], [17], [27]. On the other hand, 20% or more of people with hypertension have diabetes, and hypertension is present in up to 60% of patients with T2D [8], [9], [33], [34]. A causal relationship from hyperglycemia to hypertension is biologically plausible. Secondly, although we made careful adjustment and the sensitivity analysis showed that the association between HbA1c≥6.5% and the BP treatment target remained after excluding patients with known hypertension (thus, users of antihypertensive drugs), the confounding effects from other unmeasured factors remained possible. In this regard, behavioral factors and physical activity, which were related to hypertension control, were not collected and could not be adjusted for in our analysis. The effect sizes reported in this study may be confounded by these behavioral factors. Thirdly, our findings were obtained from patients seeking care from top tertiary hospitals in four well developed cities in China although these findings were replicated in the 4 less developed cities in China. Hence, they can not be readily extrapolated to low risk patients with T2D and further replications of these findings in other populations are needed.

In conclusion, we found that HbA1c at 6.5% was a cutoff point and a level above the cutoff point contributed to increased risk of failure to achieve the BP control goal. The findings support lowering the current hyperglycemia control goal from HbA1c<7% to 6.5%. Further follow-up studies and clinical trials are needed to confirm that tight control of hyperglycemia to a level of <6.5% can improve BP control among patients with T2D.

## Supporting Information

Table S1 Clinical and biochemical characteristics of patients with Type 2 diabetes and with diagnosed hypertension in other regional central cities in China.
(DOCX)
