# Supplementary material for: Hyperglycemia and Blood Pressure Treatment Goal: A Cross Sectional Survey of 18350 Patients with Type 2 Diabetes in 77 Tertiary Hospitals in China
Source: PLoS One. 2014 Aug 14;9(8):e103507. doi: 10.1371/journal.pone.0103507 (PMC4133202; doi:10.1371/journal.pone.0103507)
Supplement: Table S1 — Clinical and biochemical characteristics of patients with Type 2 diabetes and with diagnosed hypertension in other regional central cities in China. (DOCX) [file pone.0103507.s001.docx]

Table S1. Clinical and biochemical characteristics of patients with Type 2 diabetes and with diagnosed hypertension in other regional central cities in China

|  | BP goal not achieved  （n=8559） | BP goal achieved (n=5130) |  |
| --- | --- | --- | --- |
| Variables | Mean/number  (SD or %) | Mean/number  (SD or %) | *P* value |
| Age, year | 59.32(1.45) | 59.40(11.81) | 0.7210 |
| Male gender | 4576(53.5%) | 2531(49.3%) | <0.0001 |
| Body height, cm | 165.92(8.05) | 164.46(8.09) | <0.0001 |
| BMI, kg/m^2^ | 24.52(3.16) | 23.75(2.84) | <0.0001 |
| BMI groups, kg/m^2^ |  |  | <0.0001 |
| Overweight | 3815(44.7%) | 2063(40.3%) |  |
| Obesity | 953(11.2%) | 270(5.3%) |  |
| Duration of diabetes, year | 5.67(5.44) | 6.31(5.96) | <0.0001 |
| Duration of diabetes groups, year |  |  | <0.0001 |
| <1 year | 1283(15.0%) | 864(16.9%) |  |
| < 1-2.9 years | 2124(24.9%) | 1288(25.2%) |  |
| 3-5.9 years | 2043(24.0%) | 894(17.5%) |  |
| 6-9.9 years | 1406(16.5%) | 626(12.3%) |  |
| 10 years and above | 1672(19.6) | 1437(28.1%) |  |
| HbA1c, % | 7.99(1.77) | 7.87(1.79) | <0.0001 |
| HbA1c groups, % |  |  | <0.0001 |
| <6.0 | 543(6.3%) | 393(7.7%) |  |
| 6.0 to 6.4 | 731(8.5%) | 513(10.0%) |  |
| 6.5 to 6.9 | 1179(13.8%) | 797(15.5%) |  |
| 7.0 to 7.9 | 2605(30.4%) | 1485(28.9%) |  |
| ≥ 8.0 | 3501(40.9%) | 1942(37.9%) |  |
| Low density lipoprotein cholesterol, mmol/L | 2.76(1.17) | 2.65(0.94) | <0.0001 |
| Triglyceride, mmol/L | 2.13(1.60) | 1.97(1.28) | <0.0001 |
| Total cholesterol, mmol/L | 4.59(1.62) | 4.40(1.23) | <0.0001 |
| **Location** |  |  | <0.0001 |
| Shenyang | 4372(51.5%) | 886(17.3%) |  |
| Xi’an | 678(7.9%) | 522(10.2%) |  |
| Chengdu | 1693(19.8%) | 1894(36.9%) |  |
| Wuhan | 1816(21.2%) | 1828(35.6%) |  |

*, Median (25^th^ percentile to 75^th^ percentile) and their P values were derived from Two Sample Wilcoxon test.
